# First‐Principles Studies on Transition Metal Doped Mo2B2 as Anode Material for Li‐Ion Batteries

**DOI:** 10.1002/open.202300313

**Published:** 2024-03-05

**Authors:** Jianjian Shi, Chaojie Yu, Wei Kang, Xiuchan Xiao, Xiaoli Sun

**Affiliations:** ^1^ School of Electronic Engineering Chengdu Technological University Chengdu 611730 P. R. China; ^2^ School of Physics and Electronics Shandong Normal University Jinan 250014 P. R. China; ^3^ Beijing Graphene Institute Beijing 100095 P. R. China; ^4^ Department of Energy and Power Engineering Tsinghua University Beijing 100084 P. R. China

**Keywords:** first-principles, Mo_2_B_2_, Li-ion battery anode, two-dimensional materials

## Abstract

New two‐dimensional (2D) transition‐metal borides have attracted considerable interest in research on electrode materials for Li‐ion batteries (LIBs) owing to their promising properties. In this study, 2D molybdenum boride (Mo_2_B_2_) with and without transition metal (TM, TM=Mn, Fe, Co, Ni, Ru, and Pt) atom doping was investigated. Our results indicated that all TM‐doped Mo_2_B_2_ samples exhibited excellent electronic conductivity, similar to the intrinsic 2D Mo_2_B_2_ metal behavior, which is highly beneficial for application in LIBs. Moreover, we found that the diffusion energy barriers of Li along paths 1 and 2 for all TM‐doped Mo_2_B_2_ samples are smaller than 0.30 and 0.24 eV of the pristine Mo_2_B_2_. In particular, for 2D Co‐doped Mo_2_B_2_, the diffusion energy barriers of Li along paths 1 and 2 are reduced to 0.14 and 0.11 eV, respectively, making them the lowest Li diffusion barriers in both paths 1 and 2. This indicates that TM doping can improve the electrochemical performance of 2D Mo_2_B_2_ and that Co‐doped Mo_2_B_2_ is a promising electrode material for LIBs. Our work not only identifies electrode materials with promising electrochemical performance but also provides guidance for the design of high‐performance electrode materials for LIBs.

## Graphical abstract

Few studies have investigated whether the electrochemical performance of Mo_2_B_2_ as an LIB anode material can be improved via doping to increase the charge‐discharge rates. In this study, we successfully manipulated Mo_2_B_2_ as an LIB anode material via transition‐metal doping and screened out better TM‐doped Mo_2_B_2_, such as Co‐doped Mo_2_B_2_, to provide theoretical support.

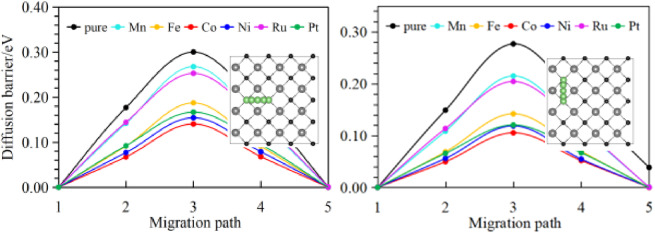

## Introduction

Since 2011, MXenes, which are two‐dimensional (2D) transition‐metal carbides/nitrides/carbon trides separated from the layered MAX phase with a general formula of M_n+1_X_n_T_x_ (n=1–4), have attracted considerable attention because of their rich configurations and adjustable characteristics.[^1^, ^2^, ^3^, ^4^, ^5^, ^6^, ^7^, ^8^, ^9^, ^10^] M, X, and T represent transition‐metal and C/N/CN_x_ surface terminations, such as O, OH, and F, respectively. Approximately 40 MXenes have been synthesized experimentally,^[10]^ and they have widespread applications in various fields such as supercapacitors,[^11^, ^12^, ^13^] secondary batteries,[^5^, ^11^, ^13^, ^14^, ^15^] catalysis,[^2^, ^10^, ^15^, ^16^, ^17^, ^18^] biomedicine,[^19^, ^20^, ^21^] electromagnetic shielding,[^22^, ^23^, ^24^, ^25^] and electronics.[^26^, ^27^, ^28^] These applications and advantages of MXenes have sparked substantial interest in the investigation of 2D transition‐metal borides (MBenes) over the past few years. Sun et al. developed a new family of 2D MBenes^[15]^ as analogs of MXenes by substituting the X element of MXenes with an adjacent B element. MBenes have the general formula M_n_B_2n‐2_ (n=2, 3, 4), where M represents the transition metal. Sun et al. discovered via first‐principles simulations that some MBenes, such as Cr_2_B_2_, Fe_2_B_2_, Mo_2_B_2_, and W_2_B_2_, could be obtained from Cr_2_AlB_2_, Fe_2_AlB_2_, MoAlB, and WAlB through selective etching. This was subsequently verified through experiments by other researchers.[^29^, ^30^] MBenes have become a large family with >50 members.^[31]^ Compared with MXenes, they have more adjustable space according to their general formula. In addition, most B‐containing compounds are high‐temperature refractory materials that exhibit metallic conductivity.[^15^, ^31^, ^32^, ^33^] Moreover, the specific surface area of MBenes is large, similar to that of MXenes, which facilitates atomic adsorption. Therefore, MBenes have received increasing attention and are expected to be used as electrode materials in Li‐ion batteries (LIBs). The theoretical gravimetric capacities of most M_2_B_2_‐type MBenes, such as Mo_2_B_2_ (444 mAh/g), Fe_2_B_2_ (665 mAh/g), V_2_B_2_ (968 mAh/g), Cr_2_B_2_ (696.1 mAh/g), and Mn_2_B_2_ (678.5 mAh/g), are higher than that of the commercial anode material graphite (372 mAh/g)[^15^, ^34^, ^35^] and the conventional Ti_3_C_2_ MXene (320 mAh/g).[^1^, ^14^] Theoretically, the diffusion energy barriers of Li are comparable to those of well‐established 2D LIB anode materials such as graphene (0.277 eV),^[36]^ MoS_2_ monolayers (0.21 eV),^[37]^ and Ti_3_C_2_O_2_ MXene (0.28 eV).^[38]^ Guo et al.^[15]^ calculated the diffusion energy barriers of Li on 2D Mo_2_B_2_ and Fe_2_B_2_ surfaces to be 0.27 and 0.24 eV, respectively. Through first‐principles studies, Jia et al.^[34]^ determined the diffusion energy barriers of Li on 2D V_2_B_2_, Cr_2_B_2_, and Mn_2_B_2_ to be 0.22, 0.28, and 0.29 eV, respectively. In a recent study, Wang et al. identified monolayer CrB, FeB, and M_n_B as highly promising anode materials for metal‐ion batteries, demonstrating a commendable rate capacity.^[39]^ Furthermore, Pan et al. made significant contributions by discovering the FeN/Fe_2_B_2_ heterojunction, which exhibited excellent electrochemical performance as an anode for LIBs.^[40]^ Additionally, Li et al. found that surface functionalization enhances the flexibility of V_4_B_6_. The functionalized material V_4_B_6_S_2_ is a promising LIB anode capable of hosting up to four layers of Li atoms. It has a high theoretical capacity of 297 mAh/g, a low diffusion barrier of 0.166 eV, and a low open‐circuit voltage of 0.136 V.^[41]^ Moreover, Mo‐based materials (such as Mo_2_B_2_, MoO_2_, and MoSe_2_) are gaining prominence as LIB anodes owing to their abundant valence states, cost‐effectiveness, and high theoretical capacity.[^42^, ^43^] Despite these advantages, certain Mo‐based anode materials face challenges, such as sluggish Li diffusion dynamics, which restrict their overall performance. For overcoming these limitations and improving the electrochemical performance for energy storage, defect engineering in MBene anode materials is regarded as a pragmatic and efficient strategy. Usually, a thin solid‐electrolyte interphase (SEI) layer is formed on the surface of the electrode during the charge‐discharge process, and Li ion diffusion inside the SEI is important and affects the overall charge transfer.[^44^, ^45^] In this study, we do not consider the effect of the SEI. Doping with Mn, Fe, Co, Ni, Ru, and Pt can be used to improve the electrochemical performance of electrode materials for LIBs.[^46^, ^47^, ^48^, ^49^, ^50^, ^51^, ^52^, ^53^] Therefore, we investigated doping with six types of transition‐metal atoms, i. e. Mn‐, Fe‐, Co‐, Ni‐, Ru‐, and Pt‐doped 2D Mo_2_B_2_, for LIB electrode materials using first principles without considering the effect of the SEI. The binding energies indicated that all six doped Mo_2_B_2_ compounds have good thermodynamic stability. Second, the adsorption energy of Li proves that the adsorption of Li atoms on 2D Mo_2_B_2_ and a transition metal (TM, TM=Mn, Fe, Co, Ni, Ru, and Pt)‐doped Mo_2_B_2_ is a spontaneous exothermic reaction, which prevents the formation of Li metal. Additionally, the density of states (DOS) suggests metallic behavior, and the diffusion energy barriers of Li are reduced, enhancing the electrochemical performance of 2D Mo_2_B_2_. Finally, all the TM‐doped Mo_2_B_2_ electrode materials exhibit better electrochemical performance than pristine Mo_2_B_2_. In summary, we identified anode materials with high electrochemical performance for LIBs. This study provides valuable guidance for designing LIB electrode materials with high electrochemical performance.

### Methodology

First‐principles calculations were performed using the Vienna Ab initio Simulation Package (VASP)^[54]^ with the plane‐wave basis set. The electron‐ion interactions and electron exchange correlation were described by the projector augmented wave method^[55]^ and generalized gradient approximation, respectively, using the Perdew‐Burke‐Ernzerhof functional. The plane‐wave basis‐set expansion was set to an energy cutoff of 500 eV. Computations for 2D Mo_2_B_2_ were based on a 4×4×1 supercell (consisting of 64 atoms). A 3×3×1 Monkhorst‐Pack^[56]^ mesh was used for the k‐point sampling of the Brillouin zone. The ionic coordinates were relaxed until the force on each atom was <0.02 eV/Å. A vacuum layer with a thickness of 15 Å was introduced to reduce the interaction between periodic images. To study the Li and Na diffusion kinetics, energy barriers were calculated using the climbing image nudged elastic band theory.^[57]^ According to the fixed initial and final structures, three images were obtained using linear interpolation.

## Results and Discussion

To evaluate whether the doped Mo_2_B_2_ could be synthesized in the experiment, the binding energies were calculated to measure the degree of aggregation or clustering of transition‐metal atoms to estimate whether a single transition metal can replace a Mo atom and bind to Mo_2_B_2_.^[58]^ Therefore, it is essential to discuss the stability of doped Mo_2_B_2_ before exploring its electrochemical performance for LIBs. The binding energies (*E*_b_) were first calculated using the following equation to measure the stability of Mo_2_B_2_ after doping. 1

(1) $$E_{b}=E_{dopant+subs}-E_{subs}-E_{bulk}$$

**Figure 1:**
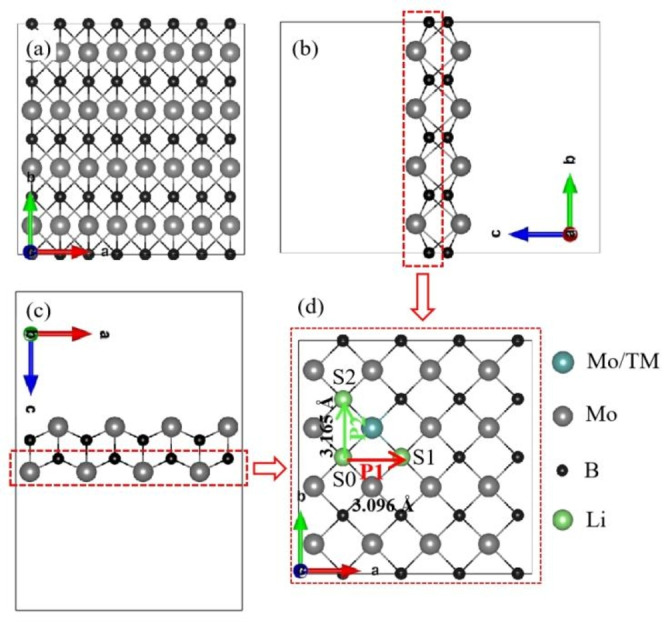
(a), (b), and (c) show the optimized Mo_2_B_2_ structures observed from three different perspectives. (d) shows a top view of the surface layer. Grey and black balls symbolize Mo and B atoms, respectively. The blue sphere represents Mo or the impurity.

Here, $E_{bulk}$ represents the total energy of one atom in the bulk, $E_{dopant+subs}\;$ represents the total energy of the doped Mo_2_B_2_, and $E_{subs}$ represents the total energy of Mo_2_B_2_ with one Mo vacancy. Provided that $E_{b}$ <0, the heteroatoms tend to be bound to Mo_2_B_2_ rather than aggregated or clustered; thus, we believe that the material satisfies the stability criterion.^[59]^ The calculated binding energies of transition metal‐doped Mo_2_B_2_ are presented in Table 1. As shown, the $E_{b\;}$ values of all the transition‐metal doped Mo_2_B_2_ samples are <0, indicating that it is energetically feasible for one transition‐metal atom to replace a Mo atom in Mo_2_B_2_. In particular, the Ni and Co dopants have lower E_b_ values than other TM dopants, i. e., −0.98 and 0.97 eV, respectivley. Moreover, we employed the projected crystal orbital Hamilton population (pCOHP) to analyze the interaction between the doped TM and the two nearest Mo atoms. This approach allows in‐depth examination of chemical bonding. As shown in Figure S1, antibonding states predominantly occur near the Fermi level for all the doped systems, suggesting relatively weak interactions between the doped TM and adjacent Mo atoms. Significantly, the antibonding orbital populations in the valence band are minimal for Mn‐doped Mo_2_B_2_, indicating stronger binding between Mn and Mo compared with the other TM‐doped Mo_2_B_2_ samples. Figure 1 shows the crystal structure of pure Mo_2_B_2_, where the black and gray balls represent B and Mo atoms, respectively. Figure 1(d) presents a top view of the surface layer for the intrinsic and TM‐doped Mo_2_B_2_. For TM‐doped Mo_2_B_2_, in Figure 1(d), where one transition metal replaces the Mo atom of pristine Mo_2_B_2_, the blue sphere represents a Mo atom or an impurity atom, and the green ball represents the adsorption site of Li.

**Table 1:** Binding energies (E_b_) of transition‐metal atoms incorporated into 2D Mo_2_B_2_ and the adsorption energies (E_ads_) of Li on the substrate.

subs	E_b_/eV	sites	*E*_ads_/eV
pure		S0	−0.69
		S1	−0.69
		S2	−0.65
Mn	−1.16	S0	−0.66
		S1	−0.66
		S2	−0.66
Fe	−0.89	S0	−0.75
		S1	−0.75
		S2	−0.75
Co	−0.97	S0	−0.76
		S1	−0.76
		S2	−0.76
Ni	−0.98	S0	−0.76
		S1	−0.76
		S2	−0.76
Ru	−0.82	S0	−0.93
		S1	−0.93
		S2	−0.93
Pt	−0.64	S0	−0.79
		S1	−0.79
		S2	−0.79

Moreover, it is important to ensure that Li atoms are firmly adsorbed on the 2D Mo_2_B_2_ material. A 4×4×1 supercell of 2D Mo_2_B_2_ was built to calculate the adsorption energy ($E_{ads}$ ) of a single Li ion on Mo_2_B_2_, which is defined as

(2) $$E_{ads}=E_{Li+subs}-E_{subs}-E_{bulk}.$$

Here,${\;E}_{Li+subs}$ , $E_{subs},\;and\;E_{bulk}$ represent the total energies of the Li‐adsorbed substrate, the substrate, and one Li atom in its body‐centered cubic bulk form, respectively. A negative adsorption energy indicates that the adsorption of Li atoms onto the substrate is a spontaneous exothermic reaction. A lower adsorption energy indicates stronger and more stable adsorption of Li on the substrate, which is beneficial for preventing the formation of Li metal and improving the safety and reversibility of LIBs. The calculated adsorption energies for Li are presented in Table 1, and the Li adsorption sites (S0, S1, and S2) are shown in Figure 1(d). As indicated by Table 1, the adsorption energy of Li on the substrates ranges from −0.93 to −0.65 eV, and all the values are <0, implying strong interactions between Li and all the substrates. Various nonequivalent positions of Li in the Mo_2_B_2_ lattice are discussed. After geometric optimization, it was observed that the surface site directly above the B atom is the most stable form for the Mo_2_B_2_, with Li−B distances of approximately 2.55 Å, and the green balls are the stable sites of Li on the Mo_2_B_2_ surface, as shown in Figure 1(d). This is consistent with the optimization results of Guo et al.. As discussed above, TM can be embedded into Mo_2_B_2_ without aggregation or clustering, whereas Li can be adsorbed on the substrate without forming metallic Li.

**Figure 2:**
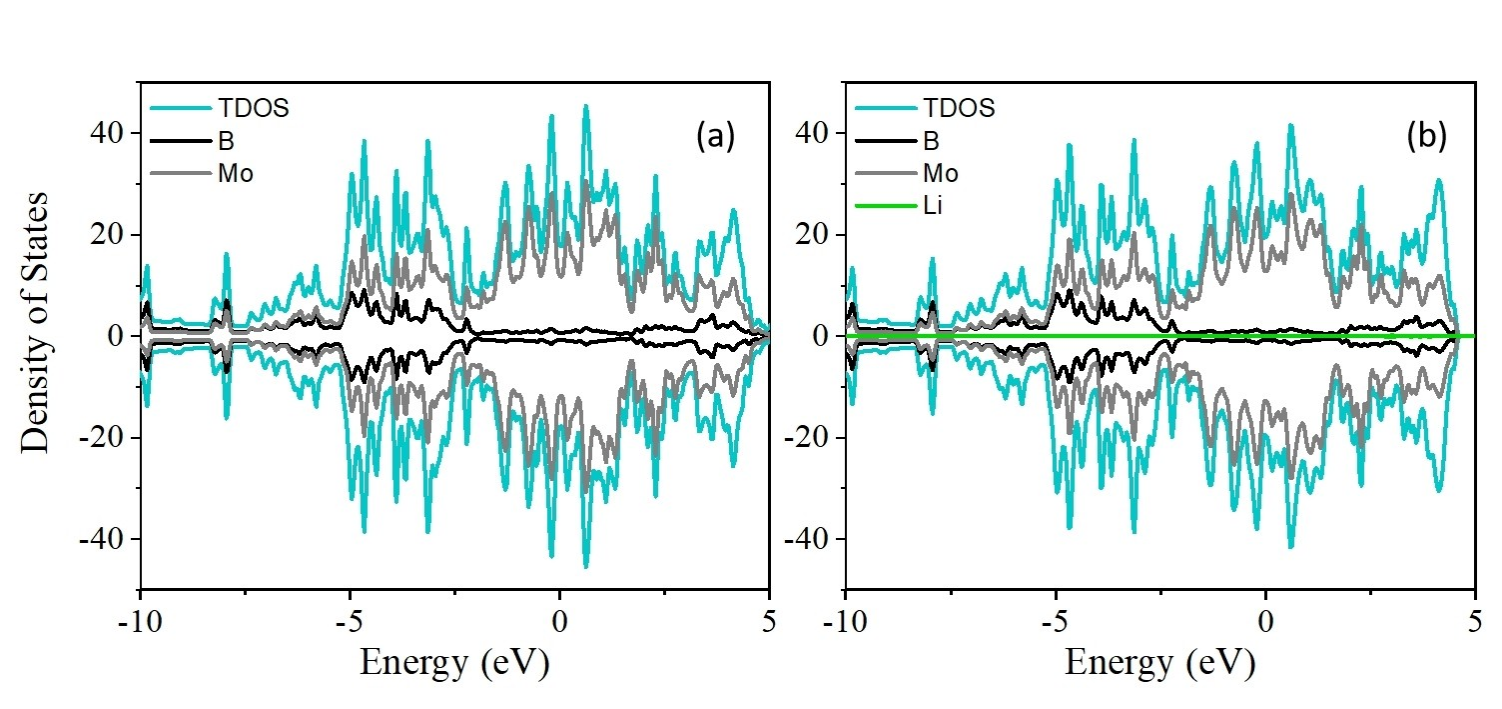
(a) and (b) show the projected DOS for pure Mo_2_B_2_ before and after Li adsorption, respectively.

**Figure 3:**
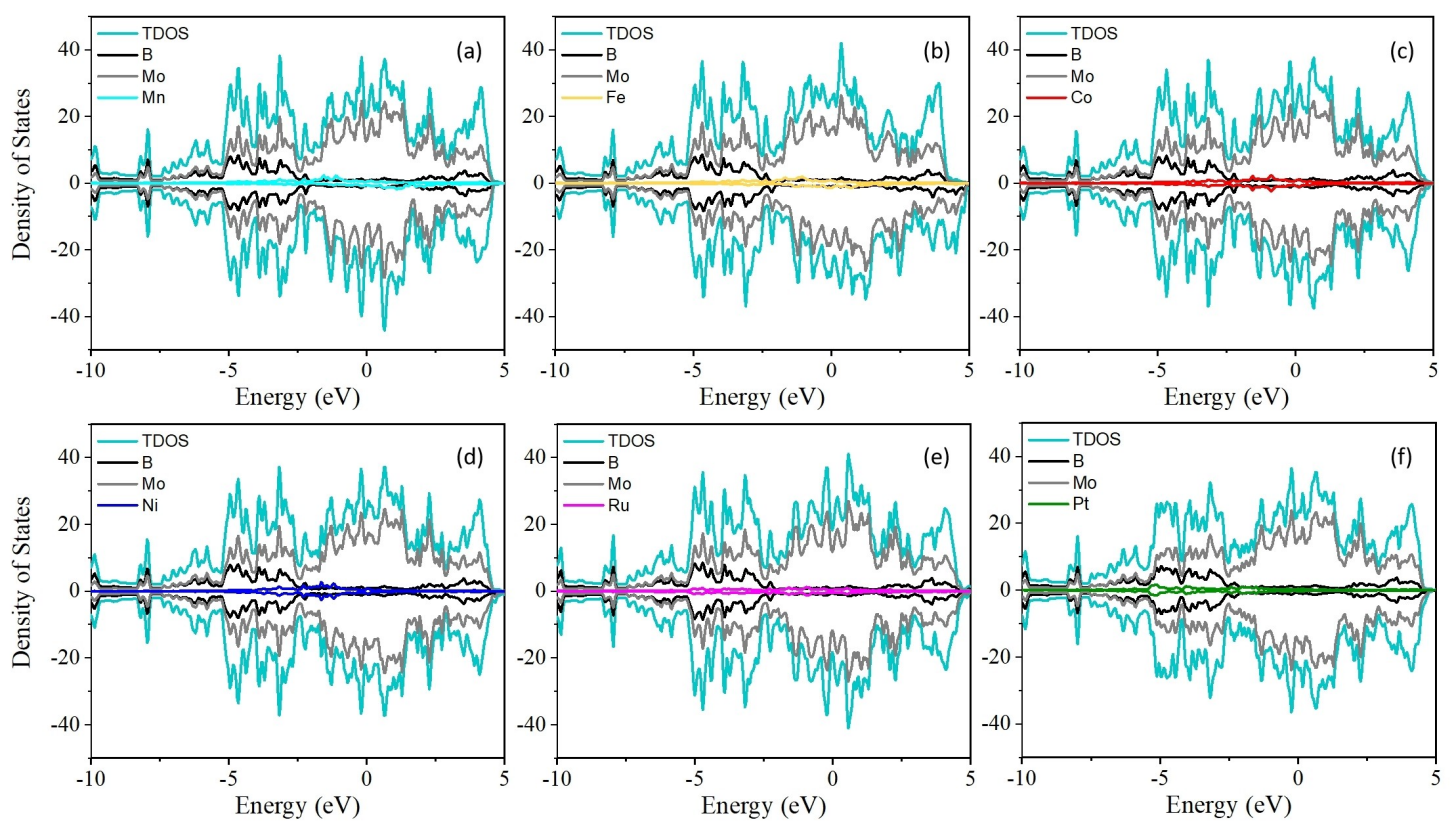
(a)–(f) show the projected DOS for TM‐doped Mo_2_B_2_.

**Figure 4:**
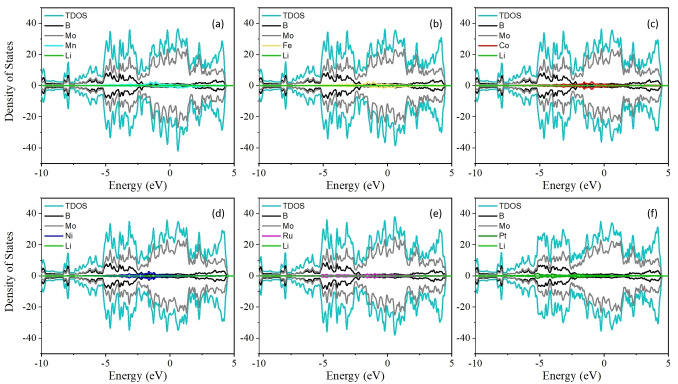
(a)–(f) show the projected DOS of TM‐doped Mo_2_B_2_ with adsorption of one Li atom.

It is well known that the conductivity and rate performance of anode materials in LIBs are closely related. Our results indicate that pristine 2D Mo_2_B_2_ and TM doped‐Mo_2_B_2_ have intrinsic metallic behavior, as shown in Figures 2(a) and 3, respectively. To obtain a deeper understanding of the electronic conductivity during the charging and discharging processes, the DOS of the original 2D Mo_2_B_2_ and TM‐doped Mo_2_B_2_ after adsorption of Li were calculated. As shown in Figure 2(b), compared with the DOS without Li adsorption, the DOS of the original 2D Mo_2_B_2_ with Li adsorbed maintained its metal conductivity during the charging process. A similar evolution of the DOS for the 2D TM‐doped Mo_2_B_2_ with Li adsorbed is shown in Figure 4; in this case, all the TM‐doped Mo_2_B_2_ compounds exhibited metallic conductivity before and after Li adsorption. These stable electronic properties during the charging and discharging processes significantly extend the potential application of TM‐doped Mo_2_B_2_ as anode materials for LIBs, and our findings can promote and guide experimental research. Moreover, we found that the orbital contribution near the Fermi level primarily comes from the substrate, but the orbital contribution of the Li atom can be ignored, which gives the system metallic properties.

**Figure 5:**
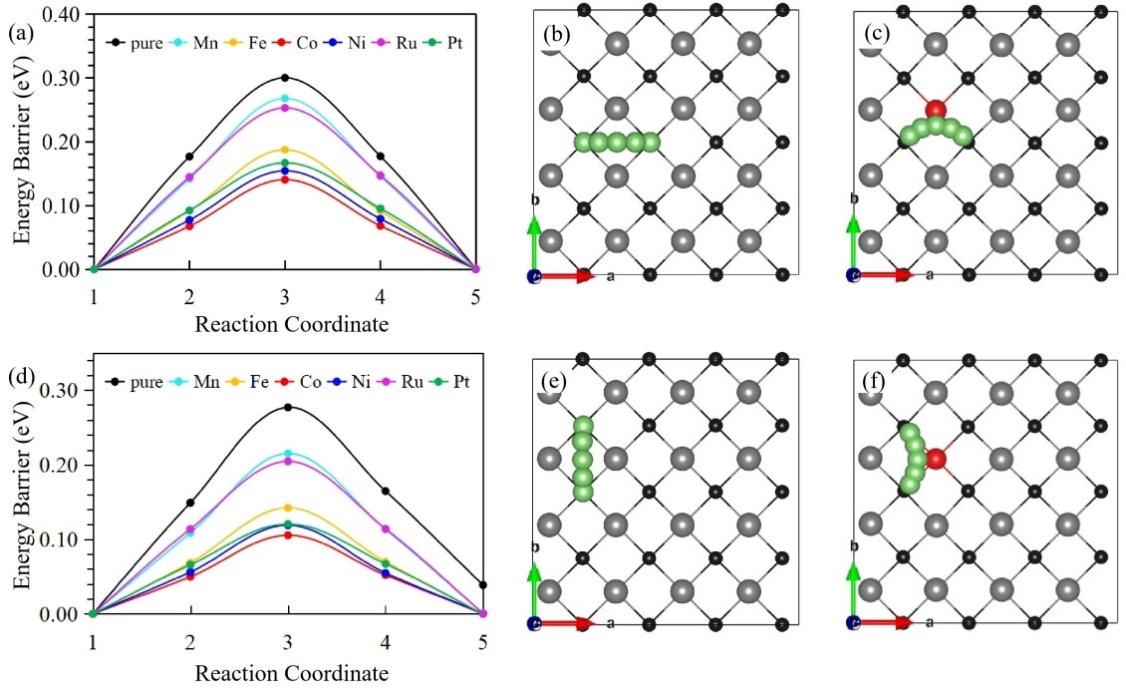
(a) shows the Li diffusion barriers for P1, while (b) and (c) show the Li diffusion paths (P1) of pure Mo_2_B_2_ and Co‐doped Mo_2_B_2_, respectively. (d) shows the Li diffusion barriers for P2, while (e) and (f) show the Li diffusion paths (P2) of pure Mo_2_B_2_ and Co‐doped Mo_2_B_2_, respectively.

For practical applications, a crucial characteristic for evaluating the suitability of an electrode material for rechargeable LIBs is the charge‐discharge rate, which depends on the diffusion behavior of the adsorbed Li atoms.^[15]^ Therefore, the electrochemical performance of pristine 2D Mo_2_B_2_ and TM‐doped Mo_2_B_2_ as promising LIBs anodes was explored in this study. The diffusion process involves the movement of Li atoms from stable positions to adjacent stable positions. As indicated by the red and green arrows in Figure 1(d), Path 1 (P1) and Path 2 (P2) are parallel to directions a and b, respectively. The distances (d) between S0 and S1 (d_s0‐s1_) and between S0 and S2 (d_s0‐s2_) are 3.096 and 3.165 Å, respectively. The diffusion energy barrier curves of Li along P1 and P2 between adjacent stable sites on the pristine 2D Mo_2_B_2_ and TM‐doped Mo_2_B_2_ surfaces are shown in Figures 5(a) and (d), respectively. For the pristine Mo_2_B_2_, the energy barriers of one Li atom along P1 and P2 are 0.30 and 0.24 eV, respectively. The corresponding migration pathways are shown in Figures 5(b) and (e). The diffusion energy barriers of Li along P1 and P2 for intrinsic 2D Mo_2_B_2_ are similar and low; the energy barrier of a Li atom along P1 is only slightly higher than that along P2 (0.06 eV). This indicates that both paths can be used for Li migration, which increases the charging and discharging rates of the LIBs. According to these results, the diffusion of Li on pristine 2D Mo_2_B_2_ is not difficult but is also not ideal. Therefore, we further investigated the Li diffusion behaviors of TM‐doped Mo_2_B_2_. As shown in Figures 5(a) and (d), the Li diffusion energy barriers along both P1 and P2 for the 2D TM‐doped Mo_2_B_2_ are lower than those for the pristine 2D Mo_2_B_2_, indicating that, all the TMs considered in this work can enhance the Li diffusion performance along both P1 and P2 of Mo_2_B_2_. For Fe‐, Co‐, Ni‐, and Pt‐doped Mo_2_B_2_, the diffusion energy barriers along P1/P2 are 0.19/0.14, 0.14/0.11, 0.15/0.12, and 0.17/0.12 eV, respectively. After doping, the diffusion energy barrier was considerably reduced, whereas there was no significant difference in the diffusion energy barrier between the two paths. The diffusion energy barrier along P2 was slightly lower, which is similar to the results for intrinsic materials. For Co‐doped Mo_2_B_2_, the Li diffusion barrier of P1 decreases from 0.30 to 0.14 eV, while the Li diffusion barrier of P2 decreases from 0.24 to 0.11 eV and is minimized at 0.11 eV, as shown in Figure 5(d), which is 0.13 eV lower than the minimum value of the pristine 2D Mo_2_B_2_. Co‐doped Mo_2_B_2_ exhibited the best electrochemical performance among the LIBs. The diffusion paths are shown in Figures 5(c) and (f). The Li diffusion barrier for the six doped Mo_2_B_2_ materials was lower than that of the original Mo_2_B_2_, and each of the six TM dopants had more valence electrons than Mo. This indicates that the electrochemical performance of Mo_2_B_2_ as an anode material for LIBs can be improved by adjusting the number of valence electrons.

To provide detailed insights into how doping influences the reduction of the Li diffusion barrier, we calculated the charge‐density differences in doped Mo_2_B_2_ both with and without Li adsorption, as illustrated in Figure S2. As shown in Figure S2(a), the dopant atoms consistently lose electrons, leading to electron gain for the B atoms. However, notable differences exist among various dopants. For example, in Co‐doped Mo_2_B_2_, the electron losses and gains along Paths 1 and 2 exhibit nearly equal distributions. In addition, the calculations revealed that the Li diffusion barriers for Co‐doped Mo_2_B_2_ along P1 are nearly identical: 0.14 and 0.11 eV, respectively. Notably, Co exhibits a larger electron loss than the other dopant atoms, which explains the low diffusion barriers observed in Co‐doped Mo_2_B_2_. Figure S2(b) shows the charge‐density differences of the doped Mo_2_B_2_ with Li adsorption, indicating that the introduction of dopants improves the charge transfer between Li and the substrates to varying degrees.

Using first principles, we found that the electronic conductivity maintained its metallic properties after doping and Li adsorption according to the DOS calculation results, and the diffusion energy barriers of Li were significantly reduced after TM (TM=Mn, Fe, Co, Ni, Ru, and Pt) doping – particularly for Fe, Co, Ni, and Pt doping. This indicates that the TM doping considered in this study can enhance the electrochemical performance of Mo_2_B_2_ as an LIB anode material.

## Conclusions

We systematically explored the electrochemical performance of a series of 2D TM‐doped Mo_2_B_2_ materials using first principles. First, we found that the binding energy confirms the stability of the TM (TM=Mn, Fe, Co, Ni, Ru, and Pt) atoms doped onto 2D Mo_2_B_2_. Second, the adsorption energy of Li indicates that Li atoms can be adsorbed on 2D Mo_2_B_2_ and TM‐doped Mo_2_B_2_ without Li aggregation or clustering. All six TM‐doped Mo_2_B_2_ samples exhibited intrinsic metallic behavior with excellent electronic conductivity and a lower diffusion energy barrier than pristine 2D Mo_2_B_2_. Co‐doped Mo_2_B_2_ had the lowest Li diffusion energy barriers of 0.14 and 0.11 eV along P1 and P2, respectively, indicating its potential for improving the electrochemical performance of LIBs. This work presents promising results on the tunable electrochemical performance of 2D TM‐doped Mo_2_B_2_ and provides valuable guidance for the design and development of LIB electrode materials with excellent electrochemical performance.

## Supporting Information

The supplementary content provided in the Supporting Information.

## Conflict of interests

There are no conflicts to declare.

1

## Supporting information

As a service to our authors and readers, this journal provides supporting information supplied by the authors. Such materials are peer reviewed and may be re‐organized for online delivery, but are not copy‐edited or typeset. Technical support issues arising from supporting information (other than missing files) should be addressed to the authors.

Supporting Information

## Data Availability

The data that support the findings of this study are available from the corresponding author upon reasonable request.
