# Supplementary material for: First‐Principles Studies on Transition Metal Doped Mo2B2 as Anode Material for Li‐Ion Batteries
Source: ChemistryOpen. 2024 Mar 5;13(8):e202300313. doi: 10.1002/open.202300313 (PMC11319228; doi:10.1002/open.202300313)
Supplement: Supplementary file 1 — Supporting Information [file OPEN-13-e202300313-s001.pdf]

ChemistryOpen

Supporting Information

First-Principles Studies on Transition Metal Doped Mo2B2 as Anode Material for Li-Ion Batteries

Jianjian Shi, Chaojie Yu, Wei Kang, Xiuchan Xiao,* and Xiaoli Sun*

Supporting Information

First-Principles Studies on Transition Metal Doped Mo_2B_2 as Anode Material for Li-Ion Batteries

Jianjian Shi,[a] Chaojie Yu,[b],[c] Wei Kang,[a] Xiuchan Xiao,*[a] and Xiaoli Sun*[c],[d]

[a] J. Shi, W. Kang, X. Xiao
School of Electronic Engineering
Chengdu Technological University
Chengdu 611730, P. R. China
E-mail: sjjian@cdtu.edu.cn; shawailsa@sina.cn

[b] C. Yu
School of Physics and Electronics
Shandong Normal University
Jinan 250014, P. R. China

[c] C. Yu, X. Sun
Beijing Graphene Institute
Beijing 100095, P. R. China
E-mail: sunxiaolideyuye@163.com

[d] X. Sun
Department of Energy and Power Engineering
Tsinghua University
Beijing, 100084, P. R. China

Methodology

The chemical bonding information by means of crystal overlap Hamilton population (COHP) was performed using the LOBSTER package.[1, 2] We also calculated the charge density differences of Mo_2B_2 after doping and Li adsorption, respectively, using the following equation,

$$\Delta\rho = \rho_{\mathrm{AB}} - \rho_{\mathrm{A}} - \rho_{\mathrm{B}} \qquad \text{(s1)}$$

where ρ_{AB} is the charge density of Mo_2B_2 after doping or Li adsorption, ρ_{A} is the charge density of Mo_2B_2 before doping or Li adsorption, ρ_{B} is the charge density of the dopant atom or the Li atom located at the same positions in the system after doping or Li adsorption. All the charge density differences were rendered using VESTA.[3]

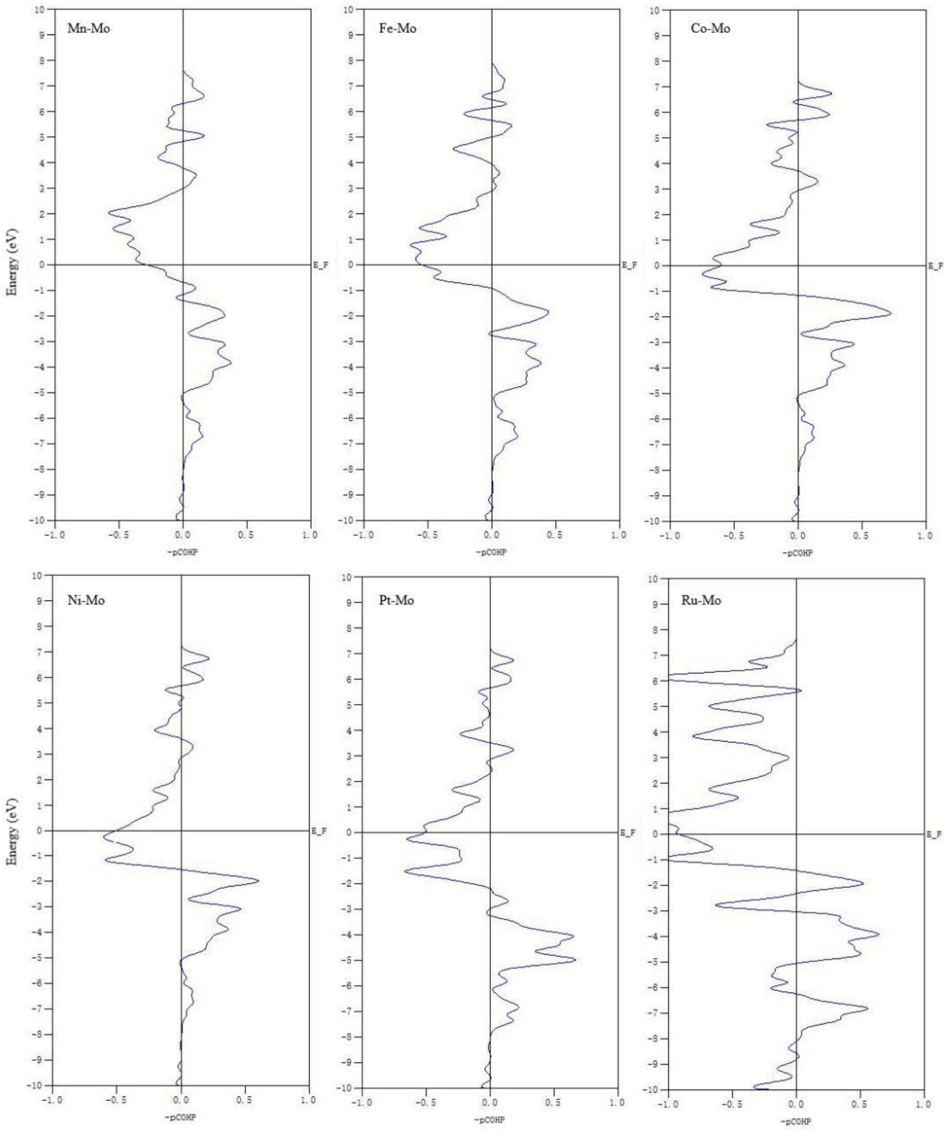

Fig. S1. Negative projected crystal orbital Hamiltonian population of TM-Mo bonds in TM-doped Mo_2B_2 without Li adsorption. Positive and negative values on the horizontal axis indicate bonding and antibonding

interactions, respectively.

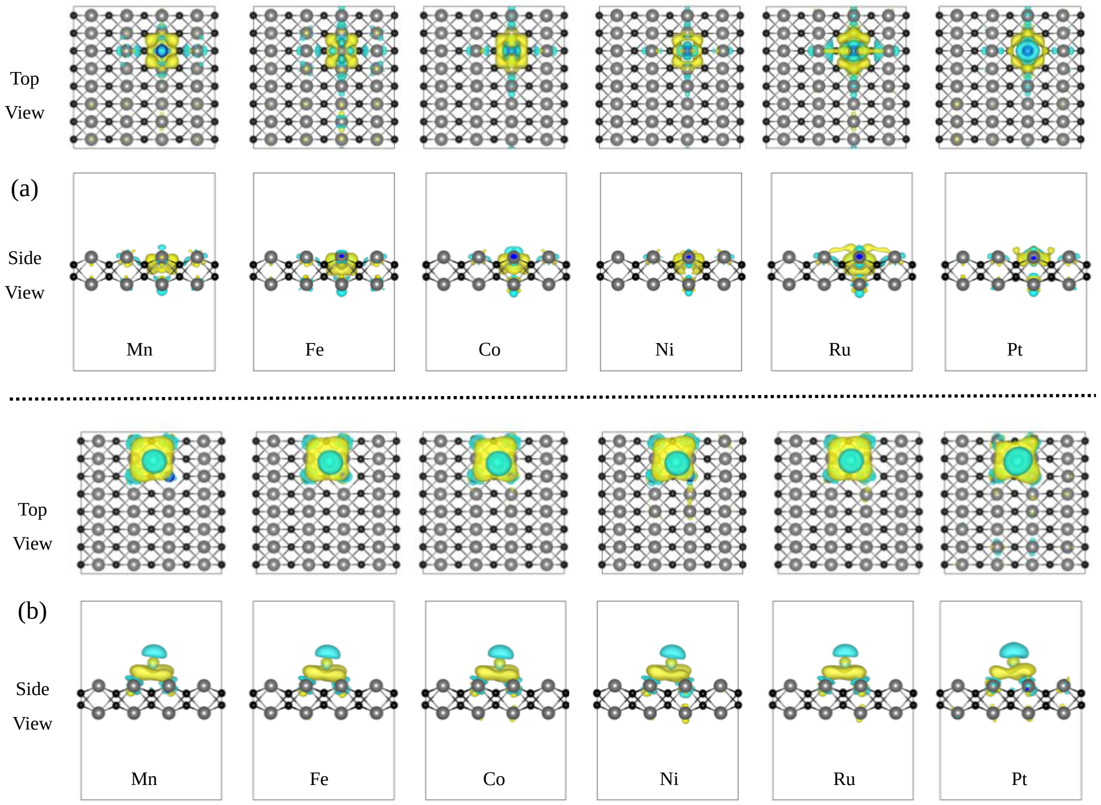

Fig. S2 Charge density difference plots (a) and (b) for TM-doped Mo_2B_2 with isosurface value 0.05 eÅ^{-3} and Li adsorbed on doped Mo_2B_2 with isosurface value 0.002 eÅ^{-3}. The yellow and cyan regions represent electron accumulation and depletion.

Reference:

[1] S. Maintz, V. L. Deringer, A. L. Tchougréeff, R. Dronskowski, Journal of Computational Chemistry 2016, 37, 1030-1035.

[2] R. Nelson, C. Ertural, J. George, V. L. Deringer, G. Hautier, R. Dronskowski, Journal of Computational Chemistry 2020, 41, 1931-1940.

[3] K. Momma, F. Izumi, Journal of Applied Crystallography 2011, 44, 1272-1276.
